# Promoting patients’ rights through hospital accreditation

**DOI:** 10.1186/s13584-020-00405-1

**Published:** 2020-09-21

**Authors:** Daniel Sperling, Rina B. Pikkel

**Affiliations:** 1 Department of Nursing, University of Haifa, Haifa, Israel; 2 International Center for Health, Law and Ethics, Faculty of Law, University of Haifa, Haifa, Israel

**Keywords:** Accreditation, patient’s rights, Rights of patient’s family, Health quality, Healthcare institutions, Regulation

## Abstract

**Background:**

Over the past decade, hospitals in many countries, including Israel, have undergone an accreditation process aimed at improving the quality of services provided. This process also refers to the protection and promotion of patients’ rights. However, reviewing the criteria and content included in this category in the Israeli context reveals definitions and implications that differ from those presented by the law – specifically the Patient’s Rights Act 1995. Moreover, the rights included in it are not necessarily equally represented in other legislation.

**Methods:**

This study seeks to examine the question of whether and to what extent the scope, contents, and definitions of patients’ rights in the JCI Standards are similar to or different from patients’ rights as they are addressed and protected in national legislation.

The article provides a comparison and examination of the different regulatory frameworks of patients’ rights, especially those in the accreditation of healthcare institution and legislation, analyzes the gaps between such frameworks, and suggests possible implications on our understanding of the concept of patients’ rights.

**Results:**

The patients’ right chapter in the accreditation process introduces and promotes the concepts of patient and family rights, increases the awareness and compliance of such concepts, and may create greater consistency in their introduction and application.

**Conclusions:**

Discussion of the Israeli case not only demonstrates how regulatory frameworks are instrumental – for broader policy purposes, especially in the area of patients’ rights and the rights of patients’ families – but also calls for a more general examination of the concept of patients’ rights in health policies and its contribution to the quality of health services. Reference to patients’ rights in accreditation of healthcare institutions may promote and enhance this concept and contribute to the delivery of care, thereby complementing a lacuna in the law.

## Background

Under international law, the enjoyment of the highest attainable standard of health is a fundamental right for every human being [1]. In this context, such a right is understood as a standard of living that is adequate for the health and wellbeing of every person and all family members [2]. Constituting an acknowledged right [3] as well as a fundamental human right [4, 5], the highest attainable standard of physical and mental health includes four basic elements: healthcare facilities and programs that (a) are adequate; (b) are equally accessible; (c) correspond with medical ethics, are sensitive towards specific communities, genders, confidentiality, and aim at improving the health status of those people concerned; and (d) are suitable for their predefined purpose, and as such are delivered together with a high level of medical and scientific quality [6]. It follows that the right to health must be understood as a right to the enjoyment of a variety of facilities, goods, services and conditions which are necessary for the realization of the highest attainable standard of health.

The term *Accreditation* relates to the process in which an external body performs an evaluation of an organization or service using a set of standards for measuring process and performance [7–9]. Per ISQua, the International Society for Quality in Healthcare, its definition includes the following keypoints: a) it is a form of external evaluation of an organization, system or programme; b) the performance of the organization, system or programme is assessed against pre-determined requirements; c) the pre-determined requirements are generally set out in standards; d) the pre-determined requirements provide a service wide approach to quality improvement focusing on both operational and clinical aspects of service provision; e) the standards may address more than legal requirement; f) assessment is undertaken by a team of reviewers from an external, independent, third party who have specific knowledge and experience of the organization, system or programme being assessed; g) the aim of accreditation is continuous quality improvement; h) a report is generated summarizing the findings from the survey, identifying areas of good practice, and providing recommendations and opportunities for improvement; i) the output of accreditation is accreditation status, namely whether it has been granted or not and the level of accreditation which has been granted; and j) accreditation status is valid for a specific and defined period [10]). Accreditation processes have been introduced into organizations and sectors around the world, including higher education institutions [11, 12], industrial fields [13], voluntary organizations [14], and health sectors [9]. The common objective of the process is to improve the quality indicators of organizations and sectors and to provide a basis for comparison – with other organizations in the same sector or among divisions and departments within the same organization [13].

Accreditation was traditionally a tool of voluntary regulation [9], conducted by independent providers upon the request of an interested organization. As a voluntary form of regulation, accreditation offers the following advantages: minimal-to-zero use of public funds and resources, increased efficiency and diminished bureaucracy, cooperation, and increased responsiveness. When carried out by an external provider, the process is often more transparent and less susceptible to political or other influences. Despite accreditation of healthcare services becoming mandatory in many parts of the world, especially the U.S. and Canada, the above listed advantages of voluntary regulation are still relevant in other fields or in countries where accreditation is yet to be obligatory.

While this type of regulatory regime offers several benefits, possible drawbacks may also be associated with the process. For example, in voluntary accreditation, available sanctions are limited and often difficult to enforce in civil or criminal law. Moreover, costs must be covered by the accredited organization which, in cases such as public hospitals, often lack sufficient resources to begin with. Another concern is that the applied standards may not necessarily fall in line with the mandatory guidelines and legislation such as those of the health system [15, 16].

In more than 100 countries including Israel, healthcare services are currently undergoing accreditation processes*.* In the context of healthcare, accreditation refers to an evaluation process carried out by an external body that examines the quality of healthcare services that are offered by healthcare organizations – usually hospitals – through standardized Quality indices [7–9, 17]. From a global perspective, up until the 1980s, this was a voluntary process designed to evaluate and adjust existing procedures and protocols to meet desired medical standards [9, 18].

Nowadays, accreditation bodies are working to create standards aimed at constantly improving the quality and safety of treatment as well as the overall management and operations of the accredited facility [19, 20]. It is believed that accreditation is a useful tool for improving healthcare service quality and safety [21]. Generally, studies worldwide note a positive effect of accreditation processes on improving aspects of management and care, such as therapeutic outcomes among patients, interactions between teams and professions, risk management, resource management, as well as internal and external standardization of care, quality and safety in treatment [17, 21–27].

Accreditation programs frequently include a chapter on the protection and promotion of patients’ rights within the healthcare organization. At times, this is regarded as part of a larger effort for assessing its performance and its organizational and ethical climate [28]. This is unique due to the central role of the patient and the potential impact of health on human rights [29]. In addition, paying attention to patients’ rights and the organizational ethics of healthcare institutions are important issues in healthcare quality [30]. Accreditation that addresses patients’ rights may also provide policymakers and patient advocacy groups with an effective instrument for informing medical practitioners, patients, and their families about such rights, and promoting and applying them within the healthcare system [31].

A range of accreditation providers offer services for healthcare organizations, the most notable ones being Accreditation Canada, the Australian Council for Healthcare Standards (ACHS) and the Joint Commission International (JCI). In the United States (USA), the Joint Commission is the primary provider of accreditation services to the healthcare sector. Since its establishment in 1951, the organization has evaluated tens of thousands of healthcare organizations and programs throughout the USA. The JCI is active in more than 40 countries, including Israel [32].

The declared goal of JCI is to improve the quality and safety of care provided by healthcare organizations around the world, through training, counseling, and service. JCI has created and implemented a set of valid standards to apply in organizations that are undergoing an accreditation process, including Patient-Centered Standards, International Patient Safety Goals (IPSG), Access to Care and Continuity of Care (ACC), Patient and Family Rights (PFR), Assessment of Patients (AOP), Care of Patients (COP), Anesthesia and Surgical Care (ASC), Medication Management and Use (MMU), Patient and Family Education (PFE), Healthcare Organization Management Standards – Quality Improvement and Patient Safety (QPS), Prevention and Control of Infections (PCI), Governance, Leadership, and Direction (GLD), Facility Management and Safety (FMS), Staff Qualifications and Education (SQE), Management of Information (MOI)), Academic Medical Center Hospital Standards – Medical Professional Education (MPE), and Human Subjects Research Programs (HRP). In order to receive a JCI certification, the hospital or other healthcare facility is required to continuously meet its standards, measures, and indicators, while undergoing re-evaluations every 3 years [19].

The Patient and Family Rights (PFR) section includes six major standards, which are further divided into the following additional standards:

- The hospital is responsible for providing processes that support the rights of patients and families during care;
- Patients are informed about all aspects of their medical care and treatment, and participate in care and treatment decision;
- The hospital informs patients and families about its process for receiving and acting on complaints, conflicts, and differences of opinion about patient care and the patients’ right to participate in these processes;
- All patients are informed about their rights and responsibilities in a manner and language they can understand;
- General consent for treatment, if obtained when a patient is admitted as an inpatient or is registered for the first time as an outpatient, is clear in its scope and limits;
- The hospital informs patients and families about how to choose to donate organs and other body tissues.

Other than the PFR section, the JCI recently initiated a patient safety campaign entitled “Speak up for your rights”, which is derived from the concept that patients have the right to be informed about their care and make related decisions. The campaign provides healthcare facilities with a range of materials to be given to their patients and families, so they may become active in their own healthcare. Launched in 2002 and updated regularly since then, this campaign includes infographics and animated videos on various topics, including anesthesia and sedation, depression, medical imaging, etc. [33].

### Accreditation of healthcare institutions in Israel; a revised model of regulation

The Israeli accreditation project began as a voluntary process, initiated by *Clalit Health Services* in 2005. As the largest healthcare organization in the country, *Clalit* implemented the process in its hospitals, with the aim of improving the quality of care provided to its insured patients. Seven years after first introducing accreditation, with all eight of *Clalitt’s* hospitals successfully receiving the JCI certificate and with reported improvement in their quality of care [29], the Ministry of Health issued an administrative guideline requiring all general hospitals in Israel to receive JCI accreditation certification – as a prerequisite for their receiving their renewed hospital license [34].

By doing so, the Ministry of Health granted the JCI official regulatory authority over the national healthcare system. As stated in the Ministry’s guidelines, this mandatory accreditation was announced “due to the advantages of working in line with valid international standards for improving the quality and safety of the care” [34]. This guideline constituted a major shift in the nature and purpose of the accreditation process in Israel from a voluntary venture initiated by one healthcare organization to an official mandatory requirement for licensing [32]. Since then, accreditation of hospitals has thus become a model of integrated regulations within the healthcare sector [35, 36]. Integrated regulation is of “A form of extrinsic motivation where identified strategies are congruent with the person own values and needs.” [37]. Since healthcare organizations aim to provide safe and high quality care, a regulation requiring that hospitals meet the standards of an external accreditor is a model of integrated regulation within health care.

While state licensing generally aims at ensuring *minimal* service standards are met by each organization, as defined by the legislator**,** the JCI accreditation process aims at ensuring *optimal* standards of service**.** This shift in the regulatory model reflects the Ministry of Health’s recognition of JCI’s optimal standards rather than sufficing with minimal standards as in the past [35]. Despite this significant shift from minimal standards to optimal standards, healthcare officials, policy makers and legislators have not addressed its potential meaning and implication so far.

Importantly, as of 2018, JCI certification has been granted to 29 general hospitals in Israel, including five Palestinian hospitals in East Jerusalem [38].

### Patients’ rights in Israeli law

The concept of patients’ rights first gained formal recognition in Israeli law in the mid-1990s, following the legislation of two main laws: The National Health Insurance Act (NHIA) in 1995, and the Patient’s Rights Act (PRA) in 1996. The NHIA specifies the fundamental principles that guide the operations and funding of the Israeli healthcare system: Justice, equality, and mutual assistance (i.e., solidarity). The law also affirmed the fundamental right to receive medical care with public funding of services that are included in the medical basket, and the consequent obligations of the state to fulfill this right [39]. The PRA then introduced a relatively elaborate bill of patients’ rights, reflecting principles previously determined in case law. These included: The right to receive medical care; the right to receive treatment without discrimination; the right to receive professional and humane treatment; the right to receive information regarding the identity of the caregivers and their role; the right to seek an additional opinion; the right to have an escort in every medical examination; the right to continuity of care; the right to medical confidentiality; the right to receive information and to review the medical records; and the right to give informed consent. The PRA also required establishment of an ethics committee in each medical institution and granted them legal authority for implementing the law and resolving possible rights-based conflicts [40, 41]. The endeavor to create a legislative patients’ rights scheme was continued in 1998 with the enactment of Equal Rights for Persons with Disabilities (ERPDL). This later developed into the right to equality in healthcare.

Over the following decade, additional rights were given formal recognition. For example, the Dying Patient Act (DPA) of 2005 and the Organ Transplant Act (OTA) of 2008 both addressed end-of-life related treatment. DPA focuses on patients’ rights during the final 6 months of their lives, acknowledging patients’ right to refuse life-prolonging treatment. While it refers to patients’ families as their proxy, this act does not view the family members as having related legal standing [42]. The OTA granted specific rights to both organ donors and their family members with regards to giving consent to the donation and establishing incentives for this purpose. While family members may still refuse to donate despite the explicit wishes of the deceased, PRA emphasizes and prioritizes the patients’ autonomy and privacy, and does not view the family members as having legal standing regarding the patients’ health and care [43–45].

Israeli courts also contributed to the establishing of patients’ rights, prior to and following the introduction of Patients’ Rights Laws. Courts developed the content of the right to healthcare [46–48], the rights of patients’ autonomy [49–51], and informed consent [49, 52, 53].

Even though the law officially protected patients’ rights since the 1990s, and successfully introduced mechanisms and tools that promote rights within the healthcare system [41, 54], there were several barriers to full implementation and enforcement of the legislation. First, doctors and other medical practitioners, were insufficiently informed [55] and were reluctant and hesitant. They perceived the law as vague [56], intervening in their clinical work [57, 58], and difficult to implement [57, 59]. Similar concerns were raised by the State Comptroller in his 2015 annual report [60]. The report reviewed the actions taken by the Ministry of Health to promote and protect patients’ rights and found them inadequate in safeguarding patients’ dignity and privacy. The report marked a disparity between hospitals as one of the main setbacks in implementing the PRA in Israel. It identified inconsistencies between healthcare providers and underlined the significance of standardizing procedures and protocols that protect patients’ rights, such as informed consent forms [60].

In summary, patients’ rights are essential for excellent healthcare performance. This is reflected in various accreditation models and reports [61]. However, to date, research has hardly focused on the implications of accreditation on patients’ rights in the healthcare system [62]. Accordingly, we have designed this research specifically to examine this important issue.

## Methods

This study examines the scope, contents, and definitions of patients’ rights in the JCI Standards and compares them to patients’ rights as they are addressed and protected in national legislation. We use the Israeli accreditation and legal system as a test case for such an examination. Specifically, we compared Israeli laws to the relevant JCI standards that are included in the PFR section.

Our methodology consists of three phases: First, we identified relevant and major legislation for acknowledging and protecting the patients’ rights currently in effect in Israel. To do this, we searched two major legal databases, Nevo and Takdinet, using the following keywords: “act” and “patients’ rights”; “health”; “medical” or “rights”.

Second, based on a literature review of textbooks and leading articles on health law and patients’ rights, we identified the protected rights and main themes in the legislation that we had found in the first phase. All of the rights, as defined in the first stage, were unequivocally stated and highlighted in the legislation searched. We then listed all the patients’ rights named in Israeli laws, the JCI standards, or both.

Third, we conducted a comparative analysis in which we critically compared the findings of the second phase, paying special attention to the scope and manner in which patient and family rights are acknowledged by the JCI and Israeli law, the role of the family, and the role of the ethics committees. This comparison demonstrates which rights are protected under the main patient rights legislation, the PRA, and which are referred to in a more specific legislation, such as the ERPL. Following this analysis, we highlighted possible future implications for policies concerning protection and promotion of patients’ rights and accreditation.

## Results

**Fig. 1:**
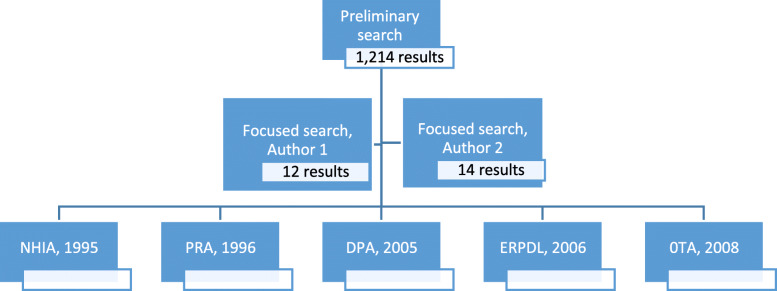
Search of Patients' rights Laws Process

Our preliminary search of major legislation for acknowledging and protecting patients’ rights in Israel, based on the keywords, yielded 1214 results. Both authors reviewed these results and screened them by relevance to the research project. One author found 12 Acts to be appropriate; the other found 14 Acts. The two authors agreed from the outset on 12 (some number less than 14). Next, the researchers discussed and agreed upon the most relevant legislation sections in all of the potentially appropriate Acts. This resulted in the following five Acts determined by the authors to be valid and applicable in Israel for specifying and protecting patients’ rights:: the National Health Insurance Act (NHA), 1995; The Patient’s Rights Act (PRA), 1997; the Dying Patient Act (DPA), 2005; The Equal Rights for Persons with Disabilities (ERPDL), 2006, and the Organ Transplant Act (OTA), 2008. Figure 1 depicts this process.

For the next phase, review of these five areas of legislation resulted in an agreed-upon list of nineteen rights of patients to be included in the comparative analysis: (1) The right to receive medical care; (2) the right to privacy; (3) the right to medical confidentiality; (4) the right to give consent; (5) the right to give informed consent; (6) the right to refuse treatment; (7) the proprietary right pertaining to the medical care; (8) the right to culture and language accommodation; (9) patients’ complaints apparatus; (10) the right to receive information; (11) the right to equality and anti-discrimination in healthcare; (12) formation of the ethics committees; (13) respect of patient’s religion; (14) the right to seek a second opinion; (15) patients’ families’ right; (16) the right to be respected/human dignity; (17) the right to be accompanied during medical examinations and treatment; (18) the right to have and receive your medical record; and (19) the right to access medical services.

Following this stage, we examined the inclusion of each of these rights, commented on it (when relevant), and mapped it in each of the five specific Acts and the JCI standards. The results of this are described in Table 1 in Appendix A.

Our comparative analysis of the various pieces of legislation and JCI standards resulted in the following findings: PRA is the primary and most important legal document that addresses the rights of patients in the Israeli healthcare system. It includes a detailed list of the rights, the first and foremost being the right to receive medical care. Such a right is interpreted as the legal recognition of the right to health in Israeli law and may serve as the basis for all other patients’ rights*.* In this bill of rights, it is clear that the legislator consciously refrains from granting any rights to the patients’ families, focusing solely on the individual patients.

Patients’ rights go beyond the scope of PRA and are protected by several other laws. The DPA deals with different issues regarding care of dying patients, namely those whose life expectancy does not exceed 6 months, including patients’ right to refuse life-sustaining treatment. It acknowledges patients’ right to comply with their families wishes in this respect. In Israel, the ODA governs organ transplants. The act regulates the way organ donations are performed and protects the donor and the recipients’ rights and interests by preventing exploitation and organ trafficking. It also directly refers to the wishes of the family as the donor’s proxy.

The PFR chapter in the JCI includes a list of standards regarding the rights of patients and their families. The chapter includes both the declaration of rights and the derivative obligations upholding them. The chapter, as well as the whole accreditation process, applies to healthcare institutions – specifically hospitals – but does not apply to providers such as sickness funds (HMOs), community clinics, and individual healthcare givers. The chapter includes rights-related standards from four categories: general standards, general consent, informed consent, and organ donation. Each category consists of main standards and measurable elements intended to broaden and clarify the standard itself.

A careful examination of JCI standards and Israeli law reveals that there are several differences in the way these regulatory frameworks deal with the issue of patients’ rights in the healthcare system. These differences can be grouped into three categories: (1) Structural differences; (2) Scope of rights; and (3) Rights of the patients’ family members.

### Structural differences

Patients’ rights in Israeli legislation are protected mostly by the PRA, with a significant focus on aspects of consent. Issues related to end of life and organ donations are covered by specific legislation. The PFR chapter includes rights established under the PRA, as well as those found in separate legislation concerning organ donation and end-of-life treatment.

It appears that the PFR chapter provides broader coverage of patients’ rights. However, the language used in the PFR chapter vs. Israeli laws covering patients’ rights differs. The JCI standards are characterized by a language of obligations and mostly refrain from making declarations or rights-related statements; whereas, all rights-related Israeli laws use a declarative language of patients’ rights – discrete from the procedures that must be employed to maintain such rights. For instance, PFR standard 5.1 entitled, “Informed Consent,” reads:“Patient informed consent is obtained through a process defined by the hospital and carried out by trained staff in a manner and language the patient can understand.” In contrast, article 13 of the PRA titled “Informed consent” reads: “No medical treatment will be provided to the patient without his informed consent, according to the provisions of this chapter.”

In summary, the PFR chapter aims at ensuring hospitals perform certain activities in line existing standards, whereas the national legislation sets the general foundations for the rights that are at stake. .

### Scope of rights

Not only does the PFR chapter protect patients’ rights that are found in a number of separate Israeli laws, but it also includes rights that are not inherently part of Israeli health legislation – including the *right to accessibility of care*. While the PRA establishes the general right to access healthcare services, it limits this right to whatever is included in existing regulations and services, as determined by the political and policymaker levels. Furthermore, Israeli law addresses matters of accessibility of care as a disability-rights issue and as part of the ERPDL. The ERPDL, like other disability rights legislation, protects the rights of disabled persons rather than the general right to access healthcare. No similar limitations are being observed under the PFR chapter, where accessibility of care is situated in a larger context and under possible obstacles, including, but not only, disability.

In addition, more than once do the JCI standards refer to patients’ cultural and religious beliefs, while related Israeli legislation does not. This may be because while in Israel consideration of different cultural and religious is applicable, the country is legally defined as a Jewish state with an Orthodox Jewish character in all areas of public administration. This definition leads to legislation prioritizing the protection of Jewish values and traditions, with less emphasis on pluralism or other religions and cultural minorities, whereas JCI standards originate from the USA and are applied worldwide, thereby necessarily addressing a large range of cultural and religious groups and needs. The influence of cultural and social factors on health accreditation systems has already been acknowledged in the literature [63].

### Rights of the patients’ families

The term “Family” is almost completely absent from the text of the PRA. The reason for this may be that the legislators deliberately avoided granting any rights or legal status to the patients’ families, aiming instead at the individual patient as having legal standing in the healthcare system. In any case, the law is influenced by a liberal philosophy emphasizing the isolated individual and her liberties vis-à-vis care providers. The law does not allow the disclosure of any patients’ information to anyone, families included, unless the patients themselves grant explicit permission to do so. While the DPA does refer to the patients’ family as their end-of-life proxy, it does not view the family as having legal standing regarding the patients’ health and care [42]. The ODA acknowledges the significance of the family in the donation process, but especially renders a right to receive relevant information.

The PFR chapter, however, choses a very different approach with regards to the rights of the patients’ family members. The acronym PFR pertains to the rights of both the patients (P) and their family (F). The family is mentioned in six different standards and thirteen measurable elements. First, in a more general and declaratory standard, it is stated that the hospital is responsible for providing processes that support the rights of the patients’ families’. In addition, the standards maintain that the hospital will support the rights of both the patients and their families to partake in the patients’ healthcare process (especially with regards to decision making). They also stipulate that the hospital should inform patients and families about their rights and responsibilities to refuse or discontinue treatment, withhold resuscitative services, and forgo or withdraw life-sustaining treatment. Next, the hospital is required to inform patients and their families about its processes for receiving and acting on complaints, conflicts, and differences of opinion regarding the patients’ care and their right to partake in these processes. Above all, patients and their families should receive adequate information about the illness, procedures, treatment, and healthcare practitioners – so that they can make education healthcare-related decisions. For the list of these standards see Table 2 in Appendix B.

It follows that these standards in the PFR provide a special and significant place for the rights of the patients’ family members in areas of informed consent, medical decision-making, and conflict resolution. This contradicts the comprehensive responsibility granted to competent individual patients about being informed and making medical decisions pertaining to their own medical condition that are mostly specified in the PRA. The PFR standards refer to the family members of a patient – not only as a proxy of the patients’ interests or as a substitute decision-maker, but also, and more meaningfully, as having legal standing: The PFR asserts that the family has the right to receive information and take part in the patients’ healthcare, as well as to be informed of possible conflicts and be involved in their resolution. In the authors’ view, this well-deserved place of the patients’ family is distinguished from that of the patient.

## Discussion

This study found assessed the possible implications of the accreditation process on patients’ rights in Israel. We examined existing laws and policies addressing patients’ rights and compared them to the standards of the JCI PFR chapter. We also paid attention to the changes in the regulatory regime of quality in healthcare and to the introduction of accreditation as part of the hospital licensing process. Three types of differences were found between the current laws in Israel and the JCI accreditation requirements that are mandated for use in Israel: Structural differences; Scope of rights; and Rights of the patients’ family members. We believe these differences lead to three types of implications: (1) Awareness and compliance; (2) (In)Consistency; and (3) Introduction of family members’ rights.

### Awareness and compliance

Despite its importance in achieving positive outcomes for patients and their families, implementing patients’ rights in Israel has encountered certain reservations and even a lack of awareness among healthcare professionals. As recent studies show, accreditation improves safety and additional indicators of healthcare quality [17, 22–25]. In addition to increasing awareness regarding patients’ rights in healthcare institutions – framed in a language of obligations – the PFR chapter of the accreditation process reaffirms the meaning of patients’ rights and consequently, the obligations of institutions and healthcare providers. Accordingly, we feel that there needs to be a government-led effort to increase the awareness and compliance with this chapter. Such an effort is likely to have a positive effect on implementing patients’ rights. It is probable that medical staff with a better understanding and attentiveness of patients’ rights will be more compliant with related standards and will be less reluctant to implement the rights of patients and their families in their practice.

The operative features of accreditation are also important. The recurrent evaluations require hospitals to prepare, train, and educate personnel on JCI standards, including the PFR chapter. The JCI instructs hospitals as to which specific actions must be taken in order to meet the standards and measurable elements and explicates how rights should be protected and promoted – thereby raising the awareness of the medical staff regarding patients’ rights.

Moreover, the integrated regulatory model of accreditation (external accreditation as a condition of state licensing) requires evaluation and training that is carried out independently, without state involvement, and is accompanied by significant enforcement measures and sanctions, specifically, revocation of a hospital’s license by the Ministry of Health. The integrated model of regulation, if enforced, is likely to enhance staff compliance with regards to promoting patients’ rights in hospitals, thereby raising efficiency rates in regulation.

### Consistency and inconsistency

Accreditation may lead to increasing both consistency and inconsistency in the protection of patients’ rights. On the one hand, the implementation of the PFR chapter as part of the accreditation process will increase consistency in the way rights are understood, protected, and promoted in healthcare institutions. In order to pass the JCI evaluation and receive accreditation, hospitals are required to generate their own procedures and forms. As rights are usually vague and declarative by nature, applying them as general principles may lead to divergence than implementing detailed standards and measurable elements. Therefore, it is reasonable to assume that accredited hospitals establish specific protocols in order to meet the necessary measurable elements. As such,.

On the other hand, while accreditation may lead to increased consistency between hospitals and regulated bodies, it may accentuate the differences between these and other healthcare organizations, such as community clinics, healthcare organizations, and independent and private physicians that are not subject to the JCI evaluation. For example, while medical personnel in hospitals should promote patients’ rights in the same standardized manner, their colleagues outside the hospital will not necessarily develop and implement a uniform standard of patients’ rights. As such, this regulatory difference can create differences and gaps in awareness of patients’ rights and overall healthcare.

### Introduction of families’ rights

The PFR chapter explicitly emphasizes the hospitals’ significant responsibility for providing processes that support the rights of patients’ families during healthcare, including providing all relevant and necessary information regarding these rights. While the Israeli legislators did not grant the patients’ families legal rights and status, the JCI standards introduce the concept of family rights and specify the measures hospitals are obliged to take in order to promote them.

Although one should inquire into to the extent with which such standards apply to and constitute an organizational culture different than the one required by the law, the incorporation of JCI standards as a mandatory licensing requirement for hospitals introduces family rights in a way that undoubtedly requires the attention of policy makers and legislators. Indeed, a direct application of the PFR chapter may contradict the main principles presented in the Israeli Bill of Patients’ Rights. Failure to provide a clear-cut policy that addresses the above-mentioned contradictions may lead to litigation that could have been prevented, as family members may seek recognition and protection of their rights in line with the hospitals’ accreditation and license requirements. One such scenario may be when a competent patient and his family are in disagreement about the best therapeutic plan for this patient. While under the PRA, the patient’s autonomy and rights prevail those of his family, implementing the PFR chapter may confer family members some legal status, thereby mandating the establishment of a balance between the parties involved or deterrence to a third party decision-making. The hospital omission or avoidance from taking such steps may, therefore raise legal action against it. A coherent policy on the status of the patients’ family in light of these different regulatory frameworks would also be beneficial, for preventing such litigation as well as for guiding medical and other hospital personnel in directing care and providing patients and families with information about their rights within the healthcare system. In light of this issue, we recommend a careful examination of each standard and measurable element is necessary to establish the desired balance between the independent evaluators’ requirements and the accepted interpretation of the current law – assuming such a balance can be achieved. Such an examination may be best achieved through a collaborative work of the Ministry of Health, the Labor, Welfare and Health Committee of the Knesset and patients’ rights organizations and advocates.

### Implication to policy-making

If addressed by policymakers, the Patients’ Rights Chapter in the accreditation process could be implemented throughout the healthcare system – not just in hospitals – with regards to informing, promoting, and applying these rights in a standardized and uniform manner. However, it should be noted that the PFR chapter may not necessarily lead to significant improvement. Thus, a study that compared between 89 hospitals in six European countries found that while accredited hospitals consistently score higher in measures of quality and safety compared to hospitals with ISO certifications or with no external assessment whatsoever, such a finding was not seen with regards to the specific dimension of Patients’ Rights [64]. Similar results were also found in a more recent study on 53 hospitals in Hungary [65].

These results are also echoed in patients’ dissatisfaction with accredited hospitals with regard to their respect of patients’ rights [66]. This may be related to the relatively limited efforts and actions required by the accreditation process on this issue, which merely focuses on posting lists of patients’ rights in corridors and rooms, or making mere declarations about the importance of such rights [67]. Such findings may reflect the challenges associated with, on the one hand, providing a comprehensive scheme by which hospitals comply with standards, and on the other hand, not putting too much burden on them in implementing these standards, especially as these standards are in some contradiction with local laws. They also reflect the fact that the accreditation process uses existing laws which are translated and conveyed to the medical team and looks for their fulfillment. Although, as shown in the ISQua definition of accreditation, the pre-determined accreditation requirements may address more than legal requirement, the process does not aim at replacing the law. These important insights therefore refer to areas which the accreditation process needs further review and re-evaluation.

Challenges regarding patients’ rights in the accreditation process should also be considered in light of the more general criticism of this process – specifically arguments warning policymakers and third-party payers not to encourage accreditation programs, as hospital accreditation might be a socially inefficient institution given the insufficient evidence of its positive outcome and due to hospital staff lack of belief in its possible quality-improvement effects [68].

Other challenges relate to the disagreement on what counts as patients’ rights, especially with regards to the healthcare institutions’ responsibilities for ensuring such rights [69] as well as the narrow focus on individual rights as opposed to healthcare justice, thereby ignoring societal issues that may affect treatment options, decision making, and quality [70].

## Conclusions

Identification of the specific Israeli laws relating to patients’ and family members’ rights in healthcare and comparison of the laws with the PFR chapter raises important issues. It suggests that the PFR chapter in the JCI accreditation process introduces and promotes the concepts of patient and family rights, increases the awareness and compliance of such concepts, and may create greater consistency in their introduction and application. Policymakers within the Ministry of Health and the Knesset but also hospital and sickness funds’ directors, professional and patient organizations and healthcare providers should address these possible implications issues and concerns so that patients’ rights will be further protected and promoted in the healthcare system. Patients merit extensive rights everywhere within the health care system. Currently, those are primarily fulfilled and promoted in the hospital sector. If policymakers extend the application of the right to accessibility of care, the right to culturally-sensitive care provision, and the rights of patient’s family to the rest of the healthcare system, then the Israeli population will be better and more equally served.

## Data Availability

All data generated or analysed during this study is publicly available.

## Appendix 1

**Table 1:** Patient rights in Israeli Legislation and JCI standard

	JCI	National Health Insurance Act	Patient Rights Act	Dying Patient Act	Equal Rights for Persons with Disabilities Law	Organ Transplant Act	Comments
**Right to Receive Medical Care**	$\underline {\mathrm{X}}$	$\underline {\mathrm{X}}$	$\underline {\mathrm{X}}$				
**Right to Privacy**	$\underline {\mathrm{X}}$	$\underline {\mathrm{X}}$	$\underline {\mathrm{X}}$		$\underline {\mathrm{X}}$*		*In ERPDL, the right to privacy is protected in all aspects of one’s life, including in receiving medical care, but not only or specifically in the medical context
**Right to Medical Confidentiality**	$\underline {\mathrm{X}}$	$\underline {\mathrm{X}}$	$\underline {\mathrm{X}}$	$\underline {\mathrm{X}}$	$\underline {\mathrm{X}}$	$\underline {\mathrm{X}}$	
**Right to Give Consent**	$\underline {\mathrm{X}}$	$\underline {\mathrm{X}}$	$\underline {\mathrm{X}}$	$\underline {\mathrm{X}}$		$\underline {\mathrm{X}}$	General consent to receive medical care
**Right to Give Informed Consent**	$\underline {\mathrm{X}}$		$\underline {\mathrm{X}}$	$\underline {\mathrm{X}}$			Informed consent to receive specific medical treatment
**Right to Refuse Treatment**	$\underline {\mathrm{X}}$	$\underline {\mathrm{X}}$*	$\underline {\mathrm{X}}$*	$\underline {\mathrm{X}}$			*In PRA and NHIA the right to refuse treatment is not mentioned, but it is implied from the right to give consent
**Proprietary Right Pertaining to the Medical Care (Protection of Patients’ Possession)**	X						Israeli law does not address this issue at all, it constitutes a gap found by comparing legislation and JCI.
**Right to Culture and Language** **accommodations**	X				X*	X**	* In ERPDL, accommodation for sign language is addressed ** In OTA, cultural and religious background of the living donor is relevant to determining the members of the donation board. **Generally –** cultural and language accommodations are the subject of a circular issued by the general manager of the Ministry of Health no. 7/11, 2011
**Patients’ Complaints Apparatus**	X	X	X		X*		* In ERPDL, complaints regarding discrimination in healthcare
**Right to Receive Information**	X		X	X		X	
**Right to Equality and Anti-Discrimination in Healthcare**		X	X		X*		** In ERPDL, regarding persons with disability
**Formation of Ethics Committees**			X	X*		X**	* In DPA, committees with similar functions (local or national committee) ** In ODA, similar function called “evaluation committee”
**Respect of Patient’s Religion**	X			X*		**	* In DPA, “This law is based on the values of the State of Israel as a Jewish and democratic state and on fundamental principles in the fields of morality, ethics and religion.” ** In ODA, religious background is relevant in choosing members of the evaluation committee
**Right to Seek a Second Opinion**	*		X				* JCI standards refer to patients’ rights within the setting of a specific hospital and not to general rights in the healthcare system
**Patients’ Families’ rights**	X*			X**		X***	* In the JCI standards, families’ rights are mentioned with regard to receiving medical information, quality and standard of care, organ donation, and complaints apparatus.
**Right to Be Respected/Human Dignity**	X*	X	X		X		* In the JCI, the hospital will respect patients’ values /beliefs. There is no mention of dignity. The right is regarded a general human right in Israeli law.
**Right to Be Accompanied During Medical Examination and Treatment**	X		X				
**Right to Have and Receive the Medical Record**	X		X	X			
**Right to Access medical Services**	X*				X**		* In JCI, including language and cultural barriers ** In ERDL, accessibility of medical facilities and services for all, especially persons with disabilities

## Appendix 2

**Table 2:** List of standards and measurable element mentioning family rights

Standard	Intent of Standard	Measurable Element	Text
PFR.1			The hospital is responsible for providing processes that support patients’ and families’ rights during care.
		PFR.1 Elements 1–3	q1. Hospital leadership works to protect and to advance patient and family rights. q 2. Hospital leadership understands patient and family rights as identified in laws and regulations and in relation to the cultural practices of the community or individual patients served. q 3. The hospital respects the right of patients, and in some circumstances the right of the patient’s family, to have the prerogative to determine what information regarding their care would be provided to family or others, and under what circumstances.
		PFR.2 Elements 1, 5–6	q1. The hospital supports and promotes patient and family participation in care processes. (Also see AOP.1.8, ME 3 and MMU.6.1, ME 4 q 5. Patients and families are informed about their right to participate in care decisions to the extent they wish. q 6. Staff members are trained on the policies and procedures and their role in supporting patient and family participation in care processes.
	Intent of PFR.1.2		When a patient or family wishes to speak with someone related to religious or spiritual needs or observe a spiritual or religious custom, the hospital has a process to respond to the request. The process may be carried out through on-site religious staff, local sources, or family-referred sources.
	Intent of PFR.1.3		Patient privacy, particularly during clinical interviews, examinations, procedures/treatments, and transport, is important. Patients may desire privacy from other staff, from other patients, and even from family members.
	Intent of PFR.1.4		The hospital communicates its responsibility, if any, for the patient’s possessions to patients and families.
	Intent of PFR.2		Patients and families participate in the care process by making decisions about care, asking questions about care, requesting a second opinion, and even refusing diagnostic procedures and treatments. For patients and families to participate in care decisions, they need basic information about the medical conditions found during assessment, including any confirmed diagnosis, and the proposed care and treatment … Patients and families understand the type of decisions that must be made about care and how to participate in those decisions. Although some patients may not wish to personally know a confirmed diagnosis or to participate in the decisions regarding their care, they are given the opportunity and can choose to participate through a family member, friend, or a surrogate decision maker… The hospital supports and promotes patient and family involvement in all aspects of care. All staff members are trained on the policies and procedures and on their role in supporting patients’ and families’ rights to participate in the care process.
PFR 2.1			The hospital informs patients and families about their rights and responsibilities to refuse or discontinue treatment, withhold resuscitative services, and forgo or withdraw life-sustaining treatments
	Intent of PFR.2.1		Some of the most difficult decisions related to refusing or withdrawing care are related to decisions about withholding resuscitative services or forgoing or withdrawing life-sustaining treatment… The hospital informs patients and families about their rights to make these decisions, the potential outcomes of these decisions, and the hospital’s responsibilities related to such decisions.
		PFR 2.1 Element 3	q 3. The hospital informs patients and families about their rights to refuse or to discontinue treatment and the hospital’s responsibilities related to such decisions.
	Intent of PFR.2.2		These needs include treatment of primary and secondary symptoms; pain management; response to the patient’s and family’s psychological, social, emotional, religious, and cultural concerns; and involvement in care decisions.
PFR.3			The hospital informs patients and families about its process to receive and to act on complaints, conflicts, and differences of opinion about patient care and the patient’s right to participate in these processes.
	Intent of PFR.3		Also, decisions regarding care sometimes present questions, conflicts, or other dilemmas for the hospital and the patient, family, or other decision makers. … The hospital has established processes for seeking resolution of such dilemmas and complaints. (Also see APR.11) The hospital identifies in policies and procedures those who need to be involved in the processes and how the patient and family participate. (Also see SQE.11)
		PFR.3 Element 4	q 4. Patients and families participate in the resolution process.
	Intent of PFR.4		The hospital prepares a written statement of patient and family rights and responsibilities that is given to patients when they are admitted as inpatients or registered as outpatients to the hospital and is available each visit or throughout their stay… The statement is appropriate to the patient’s age, understanding, and language. When written communication is not effective or appropriate, the patient and family are informed of their rights and responsibilities in a language and manner they can understand.
		PFR.5 Elements 1, 3	q 1. Patients and families are informed as to the scope of a general consent, when used by the hospital. q 3. Whether or not a general consent is obtained, all patients and families are informed about which tests and treatments require informed consent. (Also see PFR.5.1)
	Intent of PFR.5.1		Patients and families are informed as to which tests, procedures, and treatments require consent and how they can give consent. (for example, given verbally, by signing a consent form, or through some other means). Education by hospital staff is provided to patients and families as part of the process of obtaining informed consent for treatment (for example, for surgery and anesthesia). Patients and families understand who may, in addition to the patient, give consent. Designated staff members are trained to inform patients and to obtain and to document patient consent. (Also see PFR.5, ME 3 and GLD.18)
		PFR.5.1 Element 6	q 6. The identity of the individual providing the information to the patient and family is documented in the patient’s medical record.
PFR.5.3			Patients and families receive adequate information about the patient’s condition, proposed treatment(s) or procedure(s), and health care practitioners so that they can grant consent and make care decisions.
	Intent of PFR.5.3		When informed consent is not required, staff members clearly explain the proposed treatment(s) or procedure(s) to the patient and family. The information provided includes elements a) through h) as relevant to the patient’s condition and planned treatment.
PFR.6			The hospital informs patients and families about how to choose to donate organs and other tissues.
	Intent of PFR.6 and PFR.6.1		The hospital supports the choice of patients and families to donate organs and other tissues for research or transplantation. Information is provided to patients and families on the donation process and the manner in which organ procurement is organized for the community, region, or nation (such as a national or regional organ procurement agency or network) …. Hospital staff are trained on the donation process that supports patient and family choices.
		PFR.6 Elements 1–3	q 1. The hospital supports patient and family choices to donate organs and other tissues. q 2. The hospital provides information to patients and families on the donation process. q 3. The hospital provides information to the patient and family on the manner in which organ procurement is organized.

## Abbreviations

ACC: Access to Care and Continuity of Care
ACHS: The Australian Council for Healthcare Standards
AOP: Assessment of Patients
ASC: Anesthesia and Surgical Care
COP: Care of Patients
DPA: Dying Patient Act
ERPDL: Equal Rights for Persons with Disabilities Law
FMS: Facility Management and Safety
GLD: Governance, Leadership, and Direction
HRP: Human Subjects Research Programs
IPSG: International Patient Safety Goals
ISO: International Organization for Standards
ISQua: The International Society for Quality in Health Care
JCI: Joint Commission International
MMU: Medication Management and Use
MOI: Management of Information
MPE: Medical Professional Education
NHIA: National Health Insurance Act
OTA: Organ Transplant Act
PCI: Prevention and Control of Infections
PFR: Patient and Family Rights
PPE: Patient and Family Education
PRA: Patient’s Rights Act
QPS: Quality Improvement and Patient Safety
SQE: Staff Qualifications and Education
